# High-Throughput Screening of Effective siRNAs Using Luciferase-Linked Chimeric mRNA

**DOI:** 10.1371/journal.pone.0096445

**Published:** 2014-05-15

**Authors:** Shen Pang, Lauren Pokomo, Kevin Chen, Masakazu Kamata, Si-Hua Mao, Hong Zhang, Elliot Razi, Dong Sung An, Irvin S. Y. Chen

**Affiliations:** 1 Department of Orthopaedic Surgery, David Geffen School of Medicine, University of California Los Angeles, Los Angeles, California, United States of America; 2 UCLA School of Nursing, Division of Hematology-Oncology, David Geffen School of Medicine at UCLA and UCLA AIDS Institute, Los Angeles, California, United States of America; 3 Department of Molecular Genetics and Medicine and AIDS Institute, David Geffen School of Medicine, University of California Los Angeles, Los Angeles, California, United States of America; 4 School of Dentistry, University of California Los Angeles, Los Angeles, California, United States of America; 5 Department of Orthodontics, Guanghua School of Stomatology, Sun Yat-sen University, Guangzhou, China; Southern Illinois University School of Medicine, United States of America

## Abstract

The use of siRNAs to knock down gene expression can potentially be an approach to treat various diseases. To avoid siRNA toxicity the less transcriptionally active H1 pol III promoter, rather than the U6 promoter, was proposed for siRNA expression. To identify highly efficacious siRNA sequences, extensive screening is required, since current computer programs may not render ideal results. Here, we used CCR5 gene silencing as a model to investigate a rapid and efficient screening approach. We constructed a chimeric luciferase-CCR5 gene for high-throughput screening of siRNA libraries. After screening approximately 900 shRNA clones, 12 siRNA sequences were identified. Sequence analysis demonstrated that most (11 of the 12 sequences) of these siRNAs did not match those identified by available siRNA prediction algorithms. Significant inhibition of *CCR5* in a T-lymphocyte cell line and primary T cells by these identified siRNAs was confirmed using the siRNA lentiviral vectors to infect these cells. The inhibition of *CCR5* expression significantly protected cells from R5 HIV-1_JRCSF_ infection. These results indicated that the high-throughput screening method allows efficient identification of siRNA sequences to inhibit the target genes at low levels of expression.

## Introduction

RNA interference (RNAi) is a mechanism whereby small double-stranded RNA molecules of 19–25 bases in length are incorporated into the RNA-induced silencing complex (RISC) and degrade mRNA [1]–[4]. When these small inhibitory RNAs (siRNAs) are used to trigger targeted degradation of a messenger RNA, ≥90% inhibition of target transcript expression is often achievable. Thus, RNAi has tremendous potential for treating human diseases requiring reduced expression of specific genes, including using shRNA to block the expression of genes important for virus replication in human cells.

In addition to delivering synthetic double-stranded RNA into cells as siRNA, siRNAs can also be expressed from DNA or viral vectors within host cells [1], [3], [5]. Ectopically expressed forms of siRNA precursors, termed short hairpin RNAs (shRNAs), can be driven by the pol III promoters in a vector [3], [5]–[7]. The hairpin structure is recognized and cleaved by Dicer to form siRNAs that are subsequently taken up by RISC for silencing of the target mRNAs [8]. shRNAs are inverted repeats that produce an intramolecular stem-loop structure upon expression [8]. The stem structure is typically 19 to 25 bp, while the loop length varies [1]–[4]. Upon cleavage of the shRNA by Dicer, the stem provides the sense and antisense strands of the resulting *in vivo* processed siRNA. Use of synthetic siRNAs and/or vector-based shRNAs provides complementary approaches.

Although several computational methods have been proposed to identify effective shRNAs [9]–[14], there are many exceptions that do not match those predicted by the current siRNA algorithms [15]. An alternative to computer algorithms is the development of functional screening approaches. However, these require significant time and effort to quantify the efficiency of any particular shRNA clone, whether by real-time RT-PCR to quantify the degradation of mRNA or by ELISA or fluorescence-activated cell sorting (FACS) to quantify the protein that is encoded by the target mRNA [15]. As such, we developed a relatively simple luciferase-based high-throughput screening method to identify potent shRNA against C–C motif chemokine receptor 5 (CCR5) that have a potential as therapeutics for HIV disease.

We previously found that many shRNAs which were potent when expressed with the U6 promoter had much weaker activity when expressed with the H1 promoter [15]. For example, CCR5 shRNA clone 13 could efficiently inhibit the expression of CCR5 when the U6 promoter was used, but was not very efficient when the H1 promoter was used [15]. While we did successfully identify one effective shRNA, sh1005, expressed by the H1 promoter, we found that quantitative flow cytometric-based screening was extremely laborious requiring transduction and flow cytometric analysis of individual shRNA clones. As such, we set out to develop other approaches for rapid and high-throughput identification of effective CCR5 shRNAs expressed by H1. We developed an approach to quantitatively identify an efficacious shRNA against CCR5. The *CCR5* cDNA was inserted downstream of the luciferase gene (Luc). We expected that shRNA-induced mRNA degradation of the *CCR5* sequence in the Luc-CCR5 chimerical mRNA would not only destroy the *CCR5* portion of the mRNA, but also the Luc-encoding region, which is upstream of the *CCR5* gene. By quantifying the expression levels of luciferase, we were able to determine the efficiency of the shRNA clones tested.

## Materials and Methods

### Ethics statement

Human PBMC were obtained from the UCLA CFAR core facility without identification information under federal and state regulations with the approval of IRB of the University of California, Los Angeles (UCLA).

### CCR5 shRNA library

We constructed the *CCR5* library in pBluescript plasmid DNA as described in our previous study [16].

### Minipreps of CCR5 shRNA clones

We prepared the DNA of individual shRNA clones, using Qiagen 96 Turbo Miniprep plates. The *CCR5* shRNA library DNA was transformed in *E. coli* XL1-Blue strain. The *CCR5* shRNA colonies were grown in the 96-well blocks provided by the manufacturer (Qiagen, Valencia, CA) for 16 hours. The plasmid DNA was isolated according to the kit's instructions. The amounts of isolated DNA were quantified by agarose electrophoresis. Each 96-well plate contained 90 colonies from the *CCR5* library, two colonies of the positive control, pBS-H1-CCR5-1005, and four empty wells. The isolated DNA samples were saved in 96-well plates and the four empty wells were used for duplicates of both LacZ and sh1005 DNA.

### Cells and cell cultures

Human PBMC were obtained from the UCLA CFAR core facility without identification information under federal and state regulations with IRB approval. The cells were treated with PHA and IL-2 to stimulate lymphocyte proliferation and expression of *CCR5*, as previously described [15]. 293T is a SV-40 large T-antigen-transformed human embryonic kidney cell line. CEM.NKR-CCR5, provided by Dr. Alexandra Trkola [17], is a CD4+ T-lymphocyte line obtained from the National Institutes of Health AIDS reagent program (cat. 4376). Maintenance of these two cell lines was described in a prior publication [15].

### Plasmids and transfection of 293T cells

The plasmid that contains the *CCR5* cDNA downstream of the luciferase gene was constructed by inserting the *CCR5* cDNA into the pRRL-CMV-Luc plasmid, which was derived from plasmid pRRL-CMV-X-Sin [18] by replacing the GFP gene with the firefly luciferase gene. The *CCR5* cDNA from plasmid pBABE-CCR5 was obtained from the NIH AIDS reagent program (cat #3331, from Dr. Nathaniel Landau). The *CCR5* coding region was then inserted into a location downstream of the firefly luciferase gene's stop codon. The resulting plasmid, pRRL-CMV-Luc-CCR5, expressed the firefly luciferase but not the *CCR5* protein. The plasmid that contains Renilla luciferase was described previously [19]. Then, we used calcium precipitation to co-transfect 293T cells with pRRL-CMV-Luc-CCR5, most of the transfections being performed in 96-well Nunc white-wall plates (Nunc cat. no. 236108) using the reagents of the Profection kit purchased from Promega (cat. E1200). In short, 0.5 µg of pRRL-CMV-Luc-CCR5, 0.1 µg of the plasmid containing the Renilla luciferase gene, and 0.5 µg of a shRNA DNA plasmid from the CCR5 library were added into calcium precipitate transfection buffers with a final volume of 100 µl. The DNA-calcium mixture was kept at room temperature for 15 minutes. Subsequently, 10 µl of the 100 µl mixture was added into each well of 293T cells in the Nunc 96-well plates. The cell cultures were plated 24 hours prior to the transfection, with 9000 cells per well in DMEM medium supplemented with 10% fetal bovine serum, and whose medium was changed three hours prior to transfection. The wells of the Nunc-96 transfection plates contained 90 colonies from the *CCR5* shRNA library, two from a pBS-H1-CCR5-1005 miniprep, two from pBS-H1-CCR5-1005 Maxiprep, and two from pBS-H1-LacZ shRNA Maxiprep. The plasmid DNAs isolated from Maxipreps were isolated using columns purchased from Macherey-Nagel (Easton, PA). The details of transfection can be found in our attached file, Figure S1.

### Infection of CEM.NKR-CCR5 and PBMC

The FG12-based shRNA lentiviral clones were titrated by infecting 293T cells with several dilutions to determine 50% tissue culture infective doses (TCID_50_). To infect cells efficiently, the collected viral vectors were concentrated as previously described [20]. Infection of the CEM.NKR-CCR5 cell line was performed by adding viral vectors of 10^6^ TCID_50_ into wells containing 2×10^5^ cells for three hours. For infection of activated PBMC, viral vectors of 1.2×10^7^ TCID_50_ were used to infect 4×10^5^ cells.

### Monoclonal antibody, staining procedures, and flow cytometric analysis

The monoclonal antibody (mAB) used for this study was anti-huCCR5 (2D7 APC, 556903; BD Biosciences). The cells (1×10^5^) were mixed with 2 µl of anti-CCR5 mAB in 100 µl of PBS with 2% FCS, incubated at room temperature for 30 min, washed with PBS containing 2% FCS, and fixed with 2% formaldehyde in PBS. The stained cells were analyzed by FACScalibur (BD Biosciences) or Cytomics FC500 (Beckman Coulter, Fullerton, CA).

## Results

### Screening of the CCR5 shRNA library, using expression of firefly luciferase

**Figure 1:**
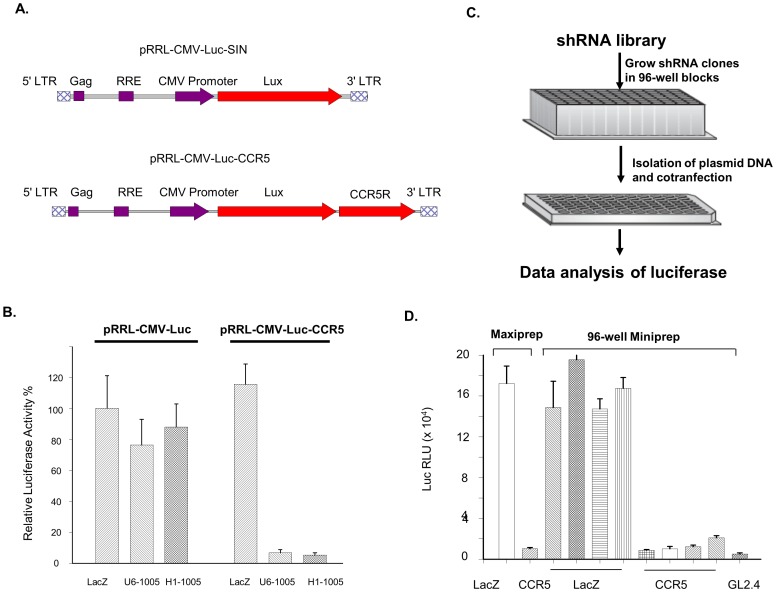
Use of the Luc-CCR5 transcript to screen CCR5 shRNA clones. A) Plasmid vectors contained either Luc alone or the Luc-CCR5 transcriptional cassette. B) Effective inhibition of luciferase expression by the sh1005 shRNA vector in pRRL-CMV-Luc-CCR5-co-transfected 293T cells. The control vector that does not contain the *CCR5* sequence did not respond to sh1005 inhibition; n = 4. C) A diagram of the *CCR5* shRNA library screening. D) sh1005 shRNA plasmid DNA isolated from 96-well Qiagen miniprep kits demonstrated inhibition similar to the same plasmid DNA isolated from Maxipreps. Four miniprep LacZ and four miniprep sh1005 DNA samples were used for transfection. Please note that clone GL2.4 is a shRNA clone specific to luciferase mRNA; n = 2.

We screened the *CCR5* shRNA library by co-expression of both the Luc-CCR5 mRNA and individual *CCR5* shRNA clones. *CCR5* cDNA was inserted into a plasmid with a pRRL backbone (Fig. 1A). The *CCR5* cDNA was inserted downstream of the luciferase gene (Luc) and upstream of the poly (A) signal sequence, so that the chimeric mRNA contains both the luciferase and CCR5 sequence. The CCR5 portion is not translated; in such a case, we focus on the target gene mRNA degradation by shRNAs and not effects on translation [21], [22]. Compared with its parental plasmid, pRRL-CMV-Luc, the constructed plasmid, pRRL-CMV-Luc-CCR5, demonstrated identical luciferase expression, indicating that inserting *CCR5* cDNA downstream of the luciferase gene's stop codon did not alter the expression profile of the Luc gene (Fig. 1B). We prepared the DNA of individual shRNA clones in 96-well plates (Qiagen 96 Turbo) as described in the Materials and Methods. Each 96-well plate contained 90 clones from the *CCR5* shRNA library, two pBS-H1-CCR5-1005 colonies, and four blanks. CCR5-1005 (sh1005) served as our positive control and is a shRNA sequence located at nts 1001 to 1020 in the CCR5 coding region. In our previous studies, this sequence demonstrated very effective at inhibiting of *CCR5* expression [16]. Following the protocol provided by the manufacturer, we collected filter-purified DNA at approximately 2 to 5 µg/well, which was dissolved in 100 µl of water. The isolated DNA samples were used to co-transfect 293T cells in a Nunc-96 white-wall plate with plasmid pRRL-CMV-Luc-CCR5 (Fig. 1C). DNA of LacZ and pBS-H1-CCR5-1005 shRNA isolated from Maxipreps were also used for co-transfections in duplicate as controls. Two days post-co-transfection, the Nunc-96 plates were assayed by a luciferase illuminator (Fig. 1C). Using miniprep pBS-H1-CCR5-1005 as a control to evaluate the quality of the DNA, we found that the plasmid DNA isolated from the 96-well plates was suitable for library screening because the sh1005 clone isolated from the 96-well plates showed inhibitory effects similar to the clone isolated from larger plasmid preps (Qiagen Maxiprep) (Fig. 1D).

**Figure 2:**
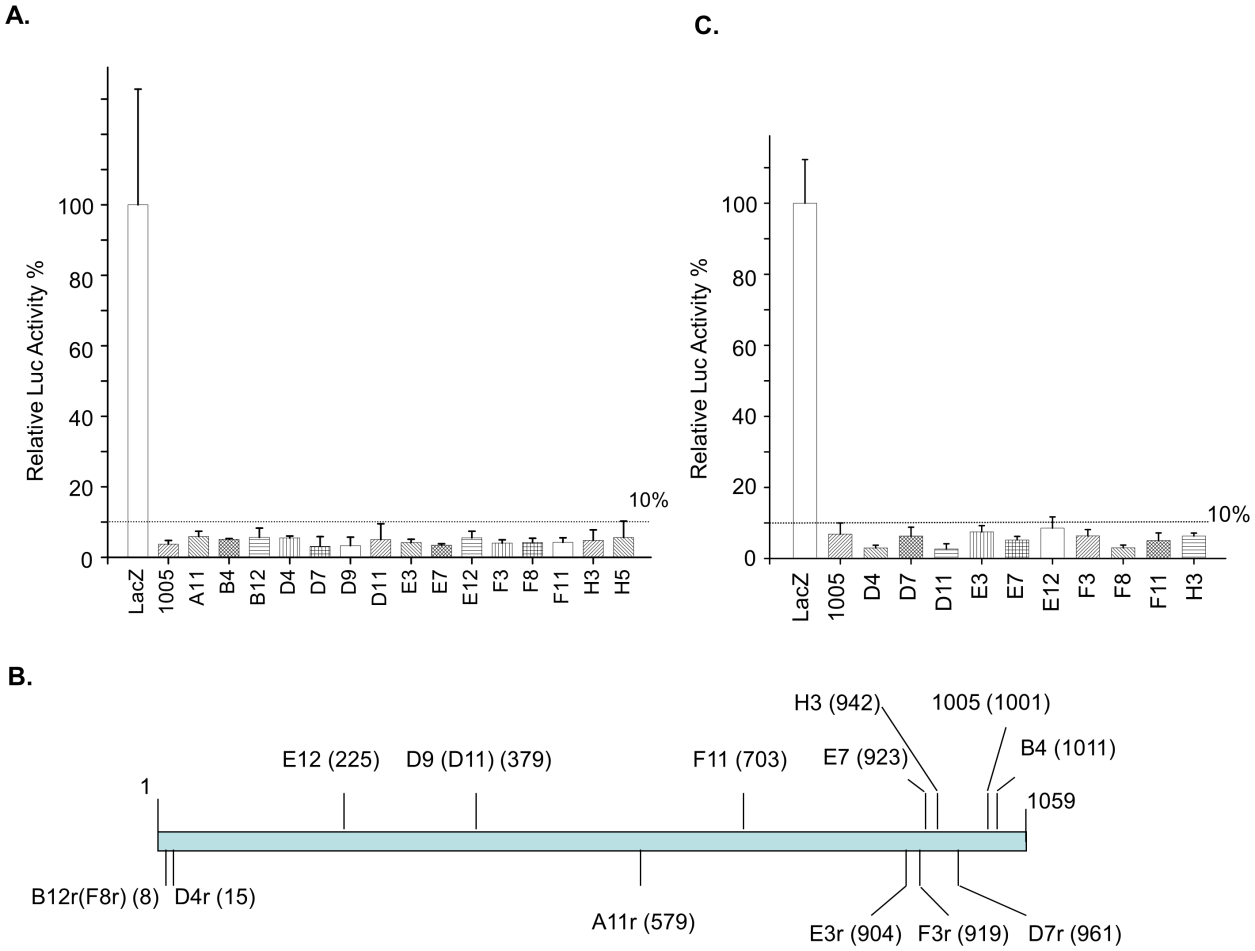
shRNA clones isolated from the second round of screening. A) 15 shRNA clones demonstrated very significant inhibition. The reference line indicates a 10% luciferase level of the LacZ control; n = 2. B) Locations of the identified shRNA clones in the CCR5 coding region. C) Confirmation of the isolated shRNA clones' ability to degrade CCR5 mRNA, as shown by reduced luciferase expression; n = 5.

In the first round of screening, we identified 81 clones that demonstrated approximately 15% luciferase activity (that is 85% inhibition) compared to the LacZ control. In most tests, the positive control, the sh1005 shRNA clone, demonstrated approximately 6% luciferase activity (94% inhibition) compared to the negative control, the pBS-H1-LacZ shRNA clone. We chose a cut-off level of 2.5 times the positive control's luciferase activity for our first round of screening, and 81 clones were isolated in the first round (Table 1). These clones were subjected to a second round of screening, and only the clones that demonstrated luciferase activity of less than 1.618-fold [23], [24] of that of the H1-sh1005 positive control were selected. The co-transfections were performed in duplicate. Using the higher cut-off level, we identified 15 shRNA clones (Table 1, Fig. 2A).

**Table 1:** shRNA clones obtained from throughput screening.

	Number of shRNA clones	Clones Obtained	Criterion	Repeats per sample
Round 1	900 clones in 10 plates	81	Luc count ≤2.5-fold Luc of sh1005 shRNA	1
Round 2	81 clones from Round 1	15	Luc count ≤1.618-fold Luc of sh1005 shRNA	2
Confirmation	15 clones from Round 2	12	Luc count ≤1.618-fold Luc of sh1005 shRNA	5

### Sequences and analysis of the identified shRNA clones

The 15 shRNA clones we identified were sequenced (Table 2), and their target sites were located (Fig. 2B). Of the 15, three were duplicates, so a total of 12 unique sequences were obtained. Of these, 10 were retested by co-transfection to confirm their inhibitory effects (Table 1, Fig. 2C).

**Table 2:** Sequences of the identified shRNA clones.

#	Name	Start	Target sequence	Commercial Match	SFOLD Score	Sense Antisense	Inhibition %
1	F8	8	ATCAAGTGTCAAGTCCAATCT	-	9	AS	97.0
2	D4	15	GTCAAGTCCAATCTATGACA	-	7	AS	97.0
3	E12	225	TGACCTGTTTTTCCTTCTTAC	-	12	S	91.4
4	D11	379	GTACCTGGCTGTCGTCCATGC	-	4	S	97.3
5	A11	579	CCAGACATTAAAGATAGTCA	-	8	AS	92.1
6	F11	704	AGGCTTATCTTCACCATCA	-	12	S	95.0
7	E3	904	GAGAAGTTCAGAAACTACC	-	7	AS	92.5
8	F3	919	TACCTCTTAGTCTTCTTCCA	-	8	AS	93.7
9	E7	923	TCTTAGTCTTCTTCCAAAAGC	-	12	S	94.8
10	H3	941	AGCACATTGCCAAACGCTTC	#Dharmacon	9	S	93.7
11	D7	961	TGCAAATGCTGTTCTATTTT	-	12	AS	93.7
12	B4	1011	AGTTTACACCCGATCCACTGG	-	13	S	93.2
13	B12*	8	ATCAAGTGTCAAGTCCAATCT	-		AS	92.4
14	H5*	8	ATCAAGTGTCAAGTCCAATCT	-		AS	92.5
15	D9*	379	GTACCTGGCTGTCGTCCATGC	-		S	95.3
16	1005	1001	GAGCAAGCTCAGTTTACACC	-	11	S	93.1

*B12 and H5 are identical to F8, and D9 is identical to D11.

# H3 is similar to Dharmacon sequence 942 except that it has an additional A in the 5′.

To determine whether the identified clones matched currently available algorithms, we performed two different modes of analysis. We first used siRNA selection programs offered by two commercial firms: Invitrogen (Carlsbad, CA), and Dharmacon (Lafayette, CO). Both the *CCR5* sequence alone and *CCR5* combined with the luciferase sequence (Luc-CCR5) were analyzed. The siRNA sequences generated from these commercial programs were compared to our shRNA sequences. We also used public web server software, SFOLD (sfold.wadsworth.org, sponsored by Wadsworth Center Health Research, Inc., Menands, NY), to analyze the predicted efficacy scores of our identified sequences in CCR5 mRNA silencing.

By comparing the siRNA sequences designed by commercial programs (not shown), we found that only one of our isolated sequences, H3, almost matched one of the siRNA sequences predicted to be efficacious by Dharmacon, except that H3 contains an extra A on the 5′ end. None of the other 11 sequences were predicted by any commercial siRNA design program. We used the SFOLD program to find the scores of our sequences. According to the algorithm of SFOLD, the siRNA scores are calculated by target accessibility, the stability of the double-stranded siRNA, the cleavage feature of the siRNA-mRNA complex in RISC, and the specificities of the siRNA designed for genes other than the target mRNA [9]. All of our sequences scored below 14 (Table 2) while there were 50 siRNA sequences with scores of 14 or higher; if we had used a computer algorithm to select siRNA sequence candidates, we would have overlooked all of these sequences when choosing shRNAs to inhibit *CCR5* expression.

### Comparison of computer program designed shRNAs with our screened CCR5 siRNA clones

**Figure 3:**
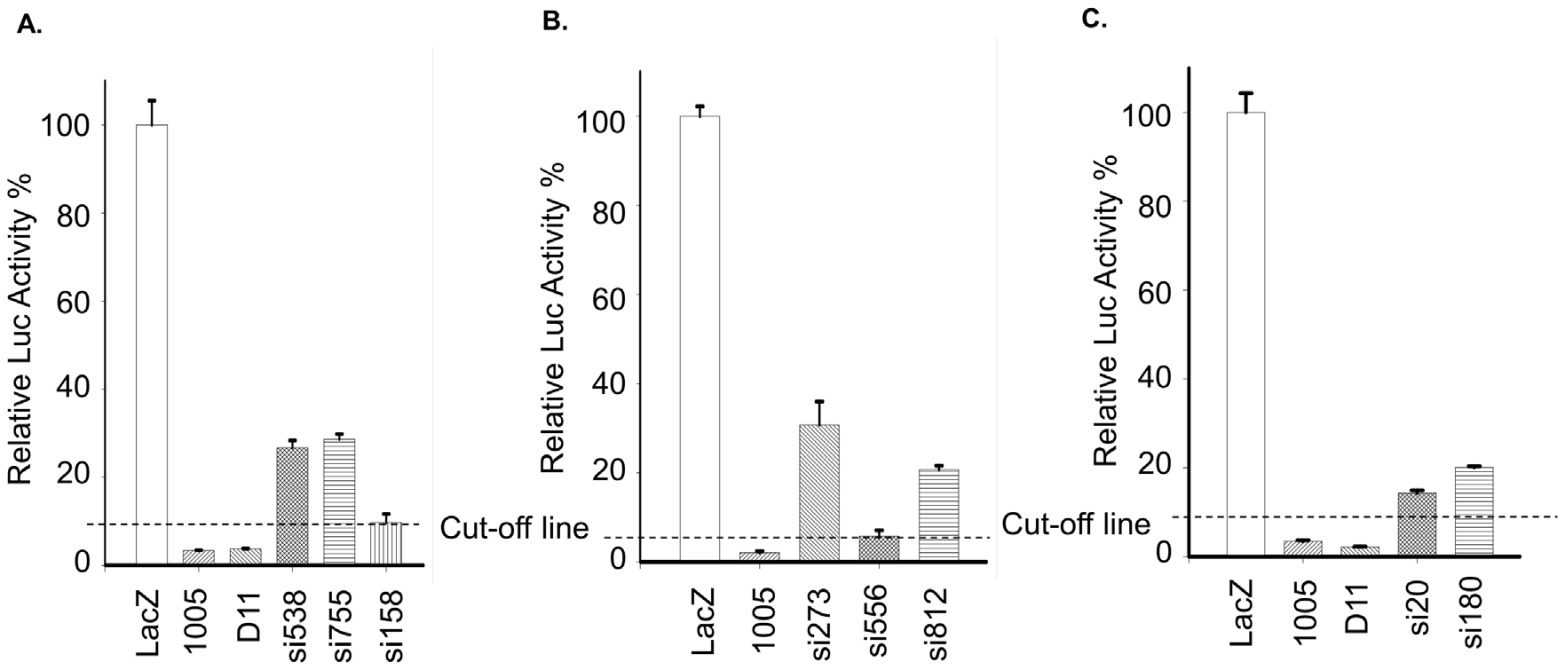
Comparison of computer program designed shRNAs with our screened CCR5 siRNA clone, sh1005. Panel A) Comparison of SFOLD sequences with sh1005. The 3 clones, at nt positions 158, 539 and 755 were scored 15, 15 and 17 by the SFOLD program were tested. The SFOLD scores of these clones were higher than the scores of both sh1005 (Score 11) and D11 (Score 4). The sequence si538 is identical to SFOLD sequence 539 except si538 has an additional T on the 5′, so we use the score of SFOLD 539 as our si538 score. As described in Results, the cut-off line represents a value 2.5 fold the luciferase activity of positive control si1005. Panel B) Comparisons of si1005 to two Dharmacon (556, 812) and two Invitrogen (273, 812) sequences in inhibition of CCR5 expression. Panel C) Comparisons of si1005 to two InvivoGen sequences (20, 180). The cut-off lines indicate the luciferase levels of 2.5× of the positive control of CCR5-1005. Results were from 293T transfection as described in Method section.

To evaluate the efficacy of high -throughput screening of an shRNA library to identify highly efficient siRNA sequences, we compared our isolated siRNAs with siRNA sequences predicted from available online algorithms. We picked two of the top rated sequences (#1 and #15) from Dharmacon, si556 and si812, two from BlockIt (Invitrogen), si273 and si812, two from siRNA Wizard (InvivoGen), and three from SFOLD (si538, si755 and si158). Although each of these sequences demonstrated some level of CCR5 inhibition, none of them met the criterion of our siRNA selection, activity resulting in less than or equal to 2.5-fold CCR5-sh1005 luciferase activity (Fig. 3). The best of these sequences was si556 from Dharmacon, which showed 2.7× luciferase activity compared to our positive control, CCR5-sh1005.

### Inhibition of *CCR5* expression in the CEM.NKR-CCR5 cell line

**Figure 4:**
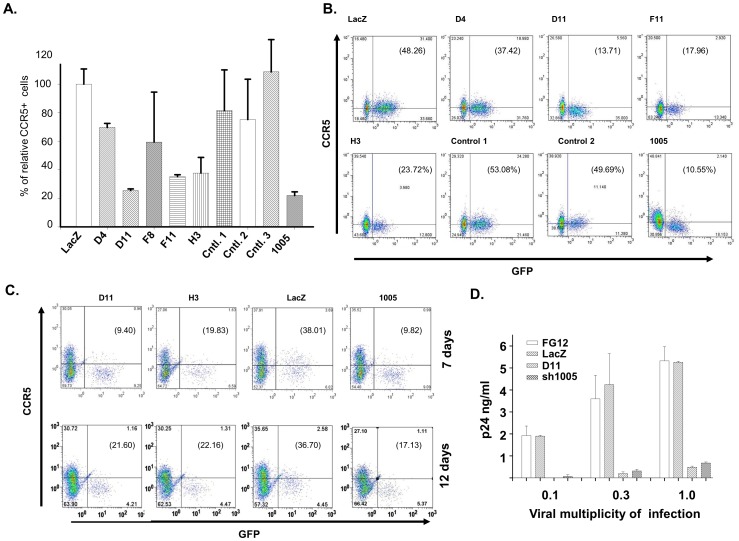
Inhibition of CCR5 expression by *CCR5* shRNA lentiviral vectors in the CEM.NKR-CCR5 cell line and activated PBL. A) Inhibition of *CCR5* expression in the CEM.NKR-CCR5 CD4-positive lymphocyte cell line, as indicated by FACS assay of CCR5-positive cells. Experiments were repeated three times. B) Examples of CCR5 inhibition by CCR5 shRNA lentiviral vectors in the CEM.NKR-CCR5 cell line. The cells that were infected by the FG12-based lentiviral vectors expressed GFP. Therefore, the GFP was used as an indicator to distinguish lentiviral vector-infected cells from the uninfected cells. C) Examples of CCR5 inhibition by CCR5 shRNA lentiviral vectors in PBL. The results of days 7 and 12 show one set, totaling three sets of shRNA vector inhibition of *CCR5* expression. The results from the other two sets either on days 7 or 12 were very similar to the results shown here. D) Inhibition of HIV-1_JRCFS_ infection by D11 shRNA vector. CEM.NKR-CCR5 cells were infected by D11, sh1005, and two controls, FG12 and FG12-LacZ. Three days post shRNA vector infection, the treated cells were subjected to HIV-1_JRCFS_ infection at the indicated titers for three hours. Subsequently, the cells were washed four times before putting in 24-well plates. At one hour of cell culture, aliquots, 100 µl, from these cell cultures were collected to check the p24 background. Three days post the infection, aliquots from the HIV-1_JRCFS_ infected cultures were collected for p24 assay.

We examined inhibition of CCR5 expression by our shRNAs in T-lymphocytes. Four identified shRNA sequences with the best inhibition of CCR5 (D4, D11, F8, and F11) and the upstream H1 promoter were inserted into plasmid pFG12 [25] to prepare corresponding lentiviral vectors. In addition, shRNA clone H3 was also selected, since this clone almost matched the siRNA sequence list when using the computer program from Dharmacon (http://www.dharmacon.com/DesignCenter). Three control shRNA clones from the CCR5 shRNA library with lower inhibitory activity were also inserted into lentiviral vectors as controls. We then used these pFG12 *CCR5* shRNA plasmids to generate lentiviral vector stocks [25] to infect the CEM.NKR-CCR5 cell line and assay inhibition of expression of *CCR5* on the cell surface. We performed FACS analysis to quantify the expression of *CCR5* at day 6 post-infection. Of the five shRNAs, three (D11, F11 and H3) demonstrated significant inhibition of *CCR5* expression (Fig. 4A, B), whereas the other two clones (D4 and F8) showed less inhibition. Additionally, the three control clones showed no inhibition, confirming our results from 293T cell transfection.

### Inhibition of CCR5 expression in activated PBMC

Using an approach similar to that for infecting the CEM.NKR-CCR5 cell line, we studied inhibition of CCR5 expression in primary blood lymphocytes by our isolated clones, D11 and H3. We selected D11 because it demonstrated the highest inhibition in CEM.NKR-CCR5 cell line and H3 was the only clone that matches the sequences from current algorithms. Our results demonstrated that the shRNA clones (D11 and 1005) strongly inhibited CCR5 expression in primary PBMC at days 7 and 12 post-infection (Fig. 4, C) while H3 shRNA demonstrated some inhibition.

### Protection of CCR5 expression cells against HIV infection by the screened shRNA clone D11

Our results demonstrated that among the screened shRNA clones, clone D11 was the most effective in silencing CCR5 expression. We expected that the inhibition of CCR5 expression could significantly inhibit the infection of R5 HIV into CCR5-dependent T cells. To test our hypothesis, we infected the CEM.NKR-CCR5 cell line with D11 as well as the control shRNA vectors. At three days post infection, the treated cells were infected with HIV-1_JRCSF_, which had various titers. The results demonstrated that D11 inhibited the infection very efficiently compared with the two negative control treatments, FG-12 lentiviral vector with no shRNA or FG-12 vector with LacZ shRNA. When a low titer of HIV virus was used, D11 treated CEM.NKR-CCR5 cells were very well protected with almost no infection (Fig. 4D). Compared with sh1005 positive control, D11 also showed stronger inhibition (Fig. 4D).

## Discussion

There are several factors involved in effective siRNA-directed sequence-specific mRNA degradation. Based on current concepts, an effective shRNA should be able to: 1) interact with RISC with high affinity, and the guide strand (antisense) of the siRNA can be correctly picked up by RISC to form a complex between siRNA and RISC; and 2) this complex binds to a particular location of the target mRNA and scans to find the correct location in the target mRNA and cleave the mRNA. Based on these concepts, several algorithms have been proposed for designing siRNA vectors. For example, commercial programs are offered by Dharmacon (http://www.dharmacon.com/DesignCenter), Invitrogen (http://rnaidesigner.invitrogen.com/rnaiexpress/design.do), InvivoGen (www.sirnawizard.com), and the noncommercial SFOLD (http://sfold.wadsworth.org).

We used the siRNA services mentioned above to identify potential siRNA sequences of *CCR5* mRNA (GenBank: U57840.1). Each of these programs provided 10 to 50 different sequences (Dharmacon, 50; Invitrogen, 10; SFOLD top 50; and InvivoGen 13). The siRNA sequence information can be found in File S1. Compared with the sequences we obtained from screening our library, none has been found when using these algorithms, although clone H3 sequence is close to that of Dharmacon sequence 942. There are several reasons to explain the difference of our results and computer algorithms, one of them being that most computer algorithms were derived from early siRNA studies and U6 promoter driven shRNAs. The difficulty of using computer programs may also be due to interactions between target mRNAs and associated proteins in cells [21], [22] and the availability of the enzymes involved in the processing of shRNA to siRNA (such as Drosha, Exportin-5 and Dicer [26], [27]) in different types of cells. Therefore, it is not surprising that none of these 12 clones, as well as the previously identified sh1005 sequences [28], matches any sequences found with these siRNA algorithms. Here, we screened a total of 900 shRNA clones to obtain 12 effective shRNAs. This yield (12/900 = 0.013) is significantly lower than shRNAs typically found to be effective when expressed with the U6 promoter (0.2–0.3). Therefore, the screening procedure described here is an approach which can be used by itself or as an adjunct with other sequence-based methods to identify rare shRNAs that are potent when expressed at reduced levels within cells. Further studies are required to understand the steps in shRNA-mediated processing, target recognition, and degradation that are favored by these particular shRNAs.

Other approaches to identify effective siRNA clones using green fluorescent protein (GFP) [29], [30], other reporter systems [31] and “bacterial invasion” [32] have been reported. However, these methods were not designed for high-throughput screening of shRNA libraries (Ref.32 is siRNA library screening). Our approach as described here is used in a 96-well format, but can be readily adapted for other plate densities. We also note the potential limitations of our screening approach. By attaching the target gene to the reporter gene, the secondary structure of the target RNA may be changed. Since the secondary structure of mRNA is important for interaction with RISC [7], [21], [33], [34], factors that can affect the secondary structure of the target mRNA [21], [22], may have an impact on the interactions between the target mRNA and RISC. In addition, the cells chosen for screening, 293T, differ from the natural cell type, primary T cells and macrophages. In this study, three of five screened shRNAs (D11, F11 and H3) demonstrated inhibition of native CCR5 expression while two of them did not show effective inhibition of CCR5. This result may be due to the above differences.

In summary, our method provides an alternative or approach to be used together with current screening methods to rapidly identify shRNAs that efficiently knockdown target gene expression.

## Supporting Information

Figure S1 **Procedure for DNA transfection.**
(PDF)

File S1 **siRNA sequences from computer algorithms.**
(PDF)
