# Supplementary material for: High-Throughput Screening of Effective siRNAs Using Luciferase-Linked Chimeric mRNA
Source: PLoS One. 2014 May 15;9(5):e96445. doi: 10.1371/journal.pone.0096445 (PMC4022502; doi:10.1371/journal.pone.0096445)
Supplement: Figure S1 — Procedure for DNA transfection. (PDF) [file pone.0096445.s001.pdf]

Figure S1

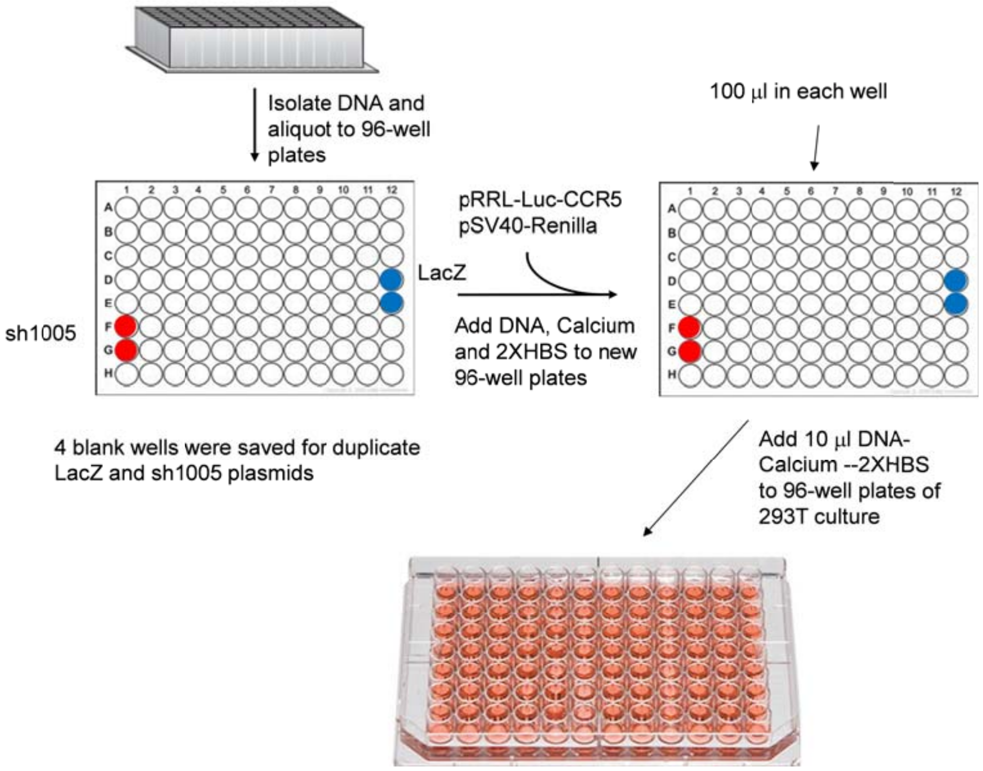

Figure S1. Procedure for DNA transfection. We prepared 90 clones of target gene shRNA library and 2 positive controls (sh1005). These miniprep DNAs were saved in a 96-well plate. Before using the DNA for transfection, plasmid DNA samples of LacZ shRNA and sh1005 vectors from Maxipreps were added into the 96-well plates. We prepared the DNA-calcium mixture in 96-well plates. We used a multichannel pipet to perform experiments. The DNA and the 2M calcium (16x) and an appropriate amount of water were mixed in the 96-well plates. Appropriate amounts of DNA, 6.25 µl 2M calcium chloride and approximately 43 µl of water were added to each well to bring the volume to 50 µl. Then 50 µl of 2x HBS were added into each well and mixed; thus each well contained 100 µl of the DNA-Calcium-HBS mixture. The mixture remained in the hood for 15 minutes before being added to the 293T cell cultures. We used 10 µl from the 100 µl of DNA-Calcium-HBS mixture to transfect 293T cells in 96-well plates.
