# Supplementary material for: High-Throughput Screening of Effective siRNAs Using Luciferase-Linked Chimeric mRNA
Source: PLoS One. 2014 May 15;9(5):e96445. doi: 10.1371/journal.pone.0096445 (PMC4022502; doi:10.1371/journal.pone.0096445)
Supplement: File S1 — siRNA sequences from computer algorithms. (PDF) [file pone.0096445.s002.pdf]

File S1

siRNA sequences generated from commercial or research algorithms

Gene Name: CCR5 Organism: Homo sapiens

Description: Human CC chemokine receptor 5 (CCR5) mRNA, complete cds.

1. Sequences from Dharmacon

Candidates: 50

Selected Sense Strand Sequence	Start Pos.	GC %	Scores
CAGTATCAATTCTGGAAGA	556	37	82
GAAATGAGAAGAAGAGGCA	674	42	82
AATGAGAAGAAGAGGCACA	676	42	81
AATAATTGCAGTAGCTCTA	799	32	81
GCCAAACGCTTCTGCAAAT	949	47	81
CAGTGGGACTTTGGAAATA	277	42	80
TATCAAGTGTCAAGTCCAA	7	37	79
CCAGTGGGACTTTGGAAAT	276	47	78
TGTCGAAATGAGAAGAAGA	670	37	78
CTGCAAATGCTGTTCTATT	960	37	78
CTGAATAATTGCAGTAGCT	796	37	77
AAGCTATGCAGGTGACAGA	830	47	76
TCATCATCCTCCTGACAAT	353	42	75
AAGAAGAGGCACAGGGCTG	682	58	75
GCTCTAACAGGTTGGACCA*	812	53	75
GTTCAGAAACTACCTCTTA	909	37	74
TCACTTGGGTGGTGGCTGT	452	58	73
AGGCTTATCTTCACCATCA	703	42	73
TGCCAAACGCTTCTGCAAA	948	47	73
GGTCATCCTCATCCTGATA	150	47	72
AGAGCATGACTGACATCTA	185	42	72
TCACCATCATGATTGTTTA	713	32	72
GAATAATTGCAGTAGCTCT	798	37	72
GCTATGCAGGTGACAGAGA	832	53	72
GCAACATGCTGGTCATCCT	140	53	71
GTCCAATCTATGACATCAA	20	37	71
GCACATTGCCAAACGCTTC	942	53	71
TGGTGTTCATCTTTGGTTT	116	37	70
TGGGCAACATGCTGGTCAT	137	53	70
TCATCCTCATCCTGATAAA	152	37	70
TGACAGGGCTCTATTTTAT	311	37	70
CTGGAATCTTCTTCATCAT	341	37	69
GCTGTGAGGCTTATCTTCA	697	47	69
GCATCAACCCCATCATCTA	872	47	69
GGGACTTTGGAAATACAAT	281	37	68
CCATGCTGTGTTTGCTTTA	393	42	68
TTAAAAGCCAGGACGGTCA	409	47	68
GGTGGTGACAAGTGTGATC	435	53	68
TTGTCATGGTCATCTGCTA	623	42	68
CGAAATGAGAAGAAGAGGC	673	47	68
GACCAAGCTATGCAGGTGA	826	53	68
TCTTAGTCTTCTTCCAAAA	923	32	68
ACTCACTGGTGTTCATCTT	110	42	67
CATCCTGATAAACTGCAAA	159	37	67
CTGACATCTACCTGCTCAA	194	47	67

ATGCTGTGTTTGCTTTAAA	395	32	67
TCATCTGCTACTCGGGAAT	632	47	67
GCAGTAGCTCTAACAGGTT	806	47	67
TGTCAAGTCCAATCTATGA	14	37	67
CCTCTTAGTCTTCTTCCAA	921	42	67

Higher Scores indicate potentially stronger inhibition.

2. Sequences from Invitrogen “Blockit” program:

No:	Start pos.	Sequences	%GC	Rank (Stars)
1	273	CGCCCAGTGGGACTTTGGAAATACA	52.0	★★★★★
2	301	TGTCAACTCTTGACAGGGCTCTATT	44.0	★★★★
3	552	CAGTCAGTATCAATTCTGGAAGAAT	36.0	★★★★
4	781	CAGGAATTCTTTGGCCTGAATAATT	36.0	★★★★★
5	812	GCTCTAACAGGTTGGACCAAGCTAT	48.0	★★★★★
6	902	GGGAGAAGTTCAGAAACTACCTCTT	44.0	★★★★
7	904	GAGAAGTTCAGAAACTACCTCTTAG	40.0	★★★★★
8	954	ACGCTTCTGCAAATGCTGTTCTATT	40.0	★★★★★
9	955	CGCTTCTGCAAATGCTGTTCTATTT	40.0	★★★★
10	1000	CGAGCAAGCTCAGTTTACACCCGAT	52.0	★★★★★

Ranked as “★★★★★” to “★★★” to indicate knockdown probability. Sequences with more stars are expected to have stronger inhibitions

3. Sequences from SFOLD program:

Start pos.	Sequences	Scores
755	ACAUUGUCCUUCUCCUGAA	17
535	AGCUCUCAUUUUCCAUACA	17
749	CCUACAACAUUGUCCUUCU	16
160	AUCCUGAUAAACUGCAAAA	16
159	CAUCCUGAUAAACUGCAAA	15
158	UCAUCCUGAUAAACUGCAA	15
30	UGACAUCAAUUAUUAUACA	15
26	UCUAUGACAUCAAUUAUUA	15
23	CAAUCUAUGACAUCAAUUA	15
773	ACACCUUCCAGGAAUUCUU	15
761	UCCUUCUCCUGAACACCUU	15

752	ACAACAUUGUCCUUCUCCU	15
750	CUACAACAUUGUCCUUCUC	15
747	UCCCUACAACAUUGUCCUU	15
539	CUCAUUUUCCAUACAGUCA	15
537	CUCUCAUUUUCCAUACAGU	15
536	GCUCUCAUUUUCCAUACAG	15
1009	UCAGUUUACACCCGAUCCA	15
1000	CGAGCAAGCUCAGUUUACA	15
909	GUUCAGAAACUACCUCUUA	14
872	GCAUCAACCCCAUCAUCUA	14
774	CACCUUCCAGGAAUUCUUU	14
770	UGAACACCUUCCAGGAAUU	14
756	CAUUGUCCUUCUCCUGAAC	14
754	AACAUUGUCCUUCUCCUGA	14
746	CUCCCUACAACAUUGUCCU	14
713	UCACCAUCAUGAUUGUUUA	14
639	CUACUCGGGAAUCCUAAAA	14
632	UCAUCUGCUACUCGGGAAU	14
550	UACAGUCAGUAUCAAUUCU	14
547	CCAUACAGUCAGUAUCAAU	14
546	UCCAUACAGUCAGUAUCAA	14
545	UUCCAUACAGUCAGUAUCA	14
541	CAUUUUCCAUACAGUCAGU	14
533	GCAGCUCUCAUUUUCCAUA	14
498	UACCAGAUCUCAAAAAGAA	14
495	CUUUACCAGAUCUCAAAAA	14
396	UGCUGUGUUUGCUUUAAAA	14
395	AUGCUGUGUUUGCUUUAAA	14
353	UCAUCAUCCUCCUGACAAU	14
344	GAAUCUUCUUCAUCAUCCU	14
341	CUGGAAUCUUCUUCAUCAU	14
340	UCUGGAAUCUUCUUCAUCA	14
218	CCAUCUCUGACCUGUUUUU	14
152	UCAUCCUCAUCCUGAUAAA	14
31	GACAUCAAUUAUUAUACAU	14
27	CUAUGACAUCAAUUAUUAU	14
22	CCAAUCUAUGACAUCAAUU	14
21	UCCAAUCUAUGACAUCAAU	14
10	CAAGUGUCAAGUCCAAUCU	14

The sequence si538 in Fig. 3 panel A is identical to sequence 539 except si538 has an additional T on the 5’.

4. Sequences from Invivogen siRNA Wizard program (www.sirnawizard.com)

Sequence	Start	GC%
GTCCAATCTATGACATCAATT	20	33.33
GGTCATCCTCATCCTGATAAA	150	42.86
GGCTGAAGAGCATGACTGACA	179	52.38
GCTGAAGAGCATGACTGACAT	180	47.62
GAAGAGCATGACTGACATCTA	183	42.86
GAGCATGACTGACATCTACCT	186	47.62
GCTTCTTCTCTGGAATCTTCT	332	42.86
GGGCTGTGAGGCTTATCTTCA	695	52.38
GGCTTATCTTCACCATCATGA	704	42.86
GCCTGAATAATTGCAGTAGCT	794	42.86
GACCAAGCTATGCAGGTGACA	826	52.38
GCTGCATCAACCCCATCATCT	869	52.38
GCTTCTGCAAATGCTGTTCTA	956	42.86
